# Investigation and Optimization of Mxene Functionalized Mesoporous Titania Films as Efficient Photoelectrodes

**DOI:** 10.3390/ma14216292

**Published:** 2021-10-22

**Authors:** Anum Iqbal, Nasser M. Hamdan

**Affiliations:** 1 Material Science and Engineering Program, The American University of Sharjah, Sharjah 26666, United Arab Emirates; g00090955@aus.edu; 2 Physics Department, The American University of Sharjah, Sharjah 26666, United Arab Emirates

**Keywords:** transition metal carbides, power conversion efficiency (PCE), surface plasmon resonance (SPR), nano structuring, photocatalyst, Mxene, photoelectrodes, Titania

## Abstract

Three-dimensional mesoporous TiO_2_ scaffolds of anatase phase possess inherent eximious optical behavior that is beneficial for photoelectrodes used for solar energy conversion applications. In this regard; substantial efforts have been devoted to maximizing the UV and/or visible light absorption efficiency; and suppressing the annihilation of photogenerated charged species; in pristine mesoporous TiO_2_ structures for improved solar illumination conversion efficiency. This study provides fundamental insights into the use of Mxene functionalized mesoporous TiO_2_ as a photoelectrode. This novel combination of Mxene functionalized TiO_2_ electrodes with and without TiCl_4_ treatment was successfully optimized to intensify the process of photon absorption; charge segregation and photocurrent; resulting in superior photoelectrode performance. The photocurrent measurements of the prepared photoelectrodes were significantly enhanced with increased contents of Mxene due to improved absorption efficiency within the visible region; as verified by UV–Vis absorption spectroscopy. The anatase phase of TiO_2_ was significantly augmented due to increased contents of Mxene and postdeposition heat treatments; as evidenced by structural analysis. Consequently; an appreciable coverage of well-developed grains on the FTO surface was observed in SEM images. As such; these newly fabricated conductive mesoporous TiO_2_ photoelectrodes are potential candidates for photoinduced energy conversion and storage applications.

## Highlights

➢ A novel Mxene functionalized Mesoporous TiO_2_ photoelectrode with a strong interconnectivity between Mxene sheets and 3D mesoporous structure is developed.
➢ Improved optical and electrical properties of conductive anatase phase 3D TiO_2_.
➢ Development of crack free films with Mxene-modified TiCl_4_ treatment.
➢ Mxene functionalized mesoporous TiO_2_ is envisaged as a valuable platform for photoinduced energy conversion and storage applications.

## 1. Introduction

Solar radiation is the most significant source of sustainable clean energy. The practical utilization of solar radiation for the generation of clean energy has been achieved through photovoltaics and by mimicking photosynthesis through the application of photocatalysis [1]. The transformation of solar illumination either into electric current or into an energy carrier is, broadly speaking, part of the domain of photoelectrochemistry [2,3]. The conversion efficacy of solar photons into charged species is mainly determined by charge separation efficiency as well as quick charge conduction toward the electrode surface, and is closely associated with the nanostructure and composition of the photoelectrode [4]. Therefore, constructing a photoelectrode capable of harvesting a broad solar spectrum has attracted widespread attention from researchers in the material sciences. The typical process which occurs at a photoelectrode surface begins with the absorption of photons by a photoresponsive layer acting as a light absorber, thus generating photogenerated charged species (e^−^/h^+^), followed by the rapid migration of these charged species towards the electrode surface [5]. Photon absorption is strongly related to the inherent extinction coefficient and nanostructure (band gap energy), which can be modulated to utilize a wide range of the solar spectrum. Furthermore, the conversion efficiency of the absorbed photons and the kinetics of the photogenerated charges are greatly influenced by the recombining centers and conductance ability of photogenerated e^−^/h^+^ within the electrode nanostructure. An extended lifetime and smaller diffusion path for phototriggered charged species facilitate quick separation and rapid conductance of charges, thus effectively improving the conversion efficacy [6]. 

The architecture of thin films to be used as photoelectrodes has been widely investigated in terms of composition, morphological features and structural attributes. These layered scaffolds possess several distinctive characteristics, e.g., a simplistic framework to anticipate and optimize the essential features of the material, particularly those associated with the dynamics of charged species in terms of their generation and charge transfer kinetics [7]. Consequently, thin films serve as an effective substrate with which to study the basic aspects of solar energy harvesting materials. After analyzing the performance of a broad range of photoactive materials, it was inferred that a unicompositional matrix fails to meet all the requirements of an effective conversion process that includes photon assimilation, suppressed recombination rate, catalytic performance and passivation [8]. Therefore, the development of a hybrid photoactive substrate composed of different chemical entities is a key approach to upgrade the efficiency of solar energy transformation reactions [9]. Each entity of the hybrid-layered structure serves a particular function, such as the light active material that should have a modulated band gap, and an electronic scaffold to maximize photon absorption within a suitable wavelength range. Furthermore, introducing cocatalysts on the top of the photoactive layer minimizes the (e^−^/h^+^) pair recombination process by facilitating quick charge separation due to close interactions between the layers. The ease of coating multiple layers of different composition on a single substratum brings the idea of a hybrid film structure into the practical realm. Various electrode material nanostructures can also be developed to enhance the process efficiency by optimizing certain desirable features [10]. 

A variety of synthetic methodologies for thin film fabrication have already been introduced, including physical vapor deposition (PVD), chemical vapor deposition (CVD), atomic layer deposition (ALD) and various other techniques, such as spin-coating and spray pyrolysis, that are based on solution precursors [11]. These solution-based techniques further strengthen the attributes of the fabricated films by enhancing the crystallinity and the strong chemical interactions between different chemical entities in a heterostructure due to postgrowth annealing treatment [12]. The thickness of the coated layer in solution-based techniques such as spin coating is mainly regulated by a uniform outward flow of fluid, which is usually considered as phase 3 of a spin coating process, and evaporation induced drying (phase 4 of a spin coating process). In order to control the thickness of a film, these two processes (i.e., viscous flow and evaporation) occur simultaneously in the coating procedure [13]. 

The highly desirable characteristics (i.e., cost effectiveness, increased chemical firmness, environmental soundness, and dominant photocatalytic attributes) of titania TiO_2_ for energy and environmental applications have been well-known for decades [14]. Among various nanostructures, the anatase nanocrystalline mesoporous architectures of TiO_2_ consisting of interlinked, nanometric-sized spheres yield an increased surface area, large pore volumes, and tunable pore sizes with unique morphologies. Furthermore, these morphological features also give rise to nanoscale effects in their mesochannels as well as on their pore walls which are greatly desired for improved performance of energy devices [15]. Increased crystallinity with fewer defects at the peripheries, without compromising the specific surface area, is an effective strategy for decreased charge annihilation processes [16]. Moreover, the combined features of increased pore size and escalated surface area intensify the adsorption of the surrounding molecules and the kinetics of electrolytic species within the mesoporous TiO_2_ framework, thus enhancing the efficiency of dye sensitized solar cells [17]. Likewise, the efficiency of perovskite solar cells can be improved by regulating the crystallinity and growing stage of the perovskite solution within the mesoporous TiO_2_ framework through the application of more intense annealing conditions [18]. The strong interaction of the perovskite matrix with the mesochannels of TiO_2_ supports the quick charge separation from the active layer, thereby improving the PCE of the device [19]. 

Generally, increased charge kinetics are desirable at the thin pore walls and shorter mesochannels [20]. Nevertheless, the conductance of photoinduced electrons within the oxide framework is supported by the diffusion process, and is predominantly affected by the charge trapping and detrapping states due to the crystallographic defects which occur within single particles and at the contact peripheries among neighboring species [21]. Along with the influence of nanoconfinement, conductive mesoporous TiO_2_ scaffolds considerably reduce the hysteresis of IV curves in the forward and reverse scanning directions. The addition of conductive species in mesoporous TiO_2_ scaffolds reduces the presence of electronic trap sites, thus enabling an enhanced electron conduction process [22]. Moreover, electron conduction within porous matrices has also been improved by fabricating porous, one-dimensional (1D) architectures composed of TiO_2_ films as photoanodes for improved PCE of perovskite-based solar devices [23]. 

A variety of materials including rare earth metals, metal oxides and carbonaceous substances have been used to dope and functionalize pristine TiO_2_ matrices. Among them, two-dimensional (2D) carbonaceous substances like graphene have attracted immense attention due to their unusual electrical conductance and suppressed recombining effects of surface (e^−^/h^+^) pairs [24,25]. Recently, transition metal carbides and nitrides, commonly called Mxenes, have emerged as candidate 2D materials. Typically, Mxenes are derived from their corresponding MAX phase starting materials through the series of chemical events shown in chemical Equations (1)–(3), where M is the transition metal (Sc, Ti, V, Cr, Zr, Nb, Mo, Hf or Ta), A belongs to IIIA or IVA group, and X could be elemental C and/or N [26].

(1) Mn+1 AXn+3HF →AF3+32 H2+Mn+1Xn

(2) $$M_{n+1}X_{n}+2H_{2}O\;\to {\;M}_{n+1}X_{n}{\left(\mathrm{OH}\right)}_{2}+{\;H}_{2}$$

(3) $$M_{n+1}X_{n}+2\mathrm{HF}\;\to {\;M}_{n+1}X_{n}F_{2}+{\;H}_{2}$$

To date, numerous Mxenes composed of various transition elements have been studied, including Ti_2_CT_x_,V_2_CT_x_, and Mo_2_CT_x_, where T signifies surface functional groups (−O, −OH, −F) [27,28]. The distinct structural phase and attached surface functionalities induce unusual characteristics in Mxene scaffolds, such as metallic conductivity, surface plasmon effects, hydrophilicity, greater surface area, and the ability to anchor a broad range of intercalates [29,30,31]. At present, most Mxenes are synthesized by etching Ti_3_AlC_2_ in HF acid due to the low reduction potential of Al [32]. The Fermi band positions of Ti_3_C_2_, O-functionalized Ti_3_C_2_, and F-functionalized Ti_3_C_2_ are −0.05, 1.88, and 0.15 V, respectively, relative to the standard hydrogen electrode (SHE), located at a lower position than the conduction band minima of TiO_2_ [33]. Therefore, the relative location of the bands in Ti_3_C_2_T_x_ manifest an appreciable transfer of photogenerated electrons from TiO_2_ matrix to Mxene, thereby reducing the chance of (e^−^/h^+^) annihilation [34]. 

Herein, TiO_2_ scaffolds are functionalized with Ti_3_C_2_ Mxene (Mx) sheets within each spin coated layer in three different steps. For the first TiO_2_:Mx layer, Mxene dispersion with a fixed volume was added to the TiO_2_ solution and then spin coated on FTO substrates through a two-step spin coating procedure. The effects of the subsequent TiCl_4_ treatment were intensified by immersing the fabricated TiO_2_:Mx films in TiCl_4_:Mx solution (with varying amounts of Mxene). Finally, the fabrication of the desired photoelectrodes was accomplished by depositing a mesoporous titania paste, with and without Mxene, through a special annealing process under ramping conditions. Therefore, this study comprises the first attempt to provide basic insights into Mxene functionalized mesoporous titania photoelectrodes in the anatase phase with improved photovoltaic features.

## 2. Materials and Methods

Titanium aluminum carbide (Ti_3_AlC_2_) powder (>98 wt% purity), hydrofluoric acid (HF) solution (40 wt%), fluorine-doped tin oxide (FTO, sheet resistance 7 Ω·sq^−1^) glass, acetone, ethanol, titanium disopropoxide bis (acetylacetonate), titanium tetrachloride solution (TiCl_4_), and mesoporous TiO_2_ commercial paste were of analytical grade. All these chemicals were purchased from Sigma Aldrich (St. Louis, MO, USA). Deionized water was utilized throughout the experimental scheme.

### 2.1. Synthesis of Mxene (Ti_3_CT_x_) Powder 

Mxene was prepared through a conventional method based upon selective etching of Ti_3_AlC_2_ powder in HF solution [35,36,37,38,39]. Commercial Ti_3_AlC_2_ powder (>98 wt% purity) was used without further purification. In a typical synthesis, 3.0 g of Ti_3_AlC_2_ powder was dissolved in HF solution (40 wt%, 50 mL). For this purpose, the HF was first poured into a plastic beaker in an ice bath. Once the HF solution had cooled to below room temperature, the desired amount of Ti_3_AlC_2_ was slowly added with continuous stirring. After the complete addition of the Ti_3_AlC_2_ over a period of 90 min, the solution was stirred under the same ice conditions for an additional 30 min. Subsequently, the solution was subjected to sonication for 2 h. During sonication, the ice water bath was refreshed after every 13 min. Finally, for the successful etching of the Al layers, the solution was kept at room temperature under stirring for 24 h. Then, the slurry was separated from HF liquid media through centrifugation at 3500 rpm for 15 min. After decanting the supernatant, the sediment was repeatedly washed with DI water by progressively increasing the centrifugation speed to 6000 rpm. When the pH was close to the desired value of 7, the sediment became more water soluble (an indication of the successful etching of Ti_3_AlC_2_ powder). In the present study, the centrifugation time was increased to 45 min at 7000 rpm for each cycle. The thoroughly washed slurry was then subjected to vacuum filtration. Finally, the Mxene powder was produced through drying the vacuum filtered cake at room temperature for further characterization. A pictorial representation of each step is presented in Figure S1 in supplementary material.

### 2.2. Fabrication of TiO_2_: Mxene Films

(a) Steps for Spin Coating: Fluorine-doped tin oxide (FTO, 7 Ω·sq^−1^) glass substrates with dimensions of 2 cm × 1.5 cm were gently cut using a glass cutter (Yucheng Technologies Ltd., Beijing, China). The FTO substrates with the required dimensions were cleaned sequentially in a sonication bath utilizing a detergent solution, deionized water, acetone, and ethanol for 30 min each, and blow-dried with an air stream. Substrates were then treated with UV-O_3_ for 40 min to remove any organic contaminants. Figure S2 in supplementary material illustrates the stages involved in the fabrication of Mxene-modified, mesoporous titania photoelectrodes. A thin compact TiO_2_:Mxene scaffold was spin coated on the required FTO sizes through a two-step process: (1) spinning at 4000 rpm for 20 s just after dropping down the required solution, followed by (2) immediate drying at 8000 rpm for 10 s. A high spinning speed causes a thinning of the layer with uniform evaporation of the solvent. Highly volatile chemical entities in the deposited solution can be removed with a high spinning speed, but compounds of low volatility remain on the surface of the substrate [40]. Therefore, the coated films were further dried at 120 °C for 5 min on a hotplate. The dried films were then annealed at 500 °C for 30 minutes in ambient air. The as prepared films were then subjected to Mxene modification and simple TiCl_4_ treatment, respectively.

(b) Preparation of Mxene:TiO_2_ precursor solution: The precursor solution for the TiO_2_:Mxene films was prepared by adding 0.586 mL of Mxene dispersion (0.5 mg/mL in ethanol) and 0.586 mL of titanium disopropoxide bis (acetylacetonate) (99.9% Sigma-Aldrich, St. Louis, MO, USA) to 3.414 mL of ethanol. The prepared solution was stirred for 10 min. Then, 110 µL of this solution was dropped onto the FTO substrates.

(c) Mxene-modified TiCl_4_ treatment: The TiO_2_:Mx films were then subjected to TiCl_4_ treatment with or without Mxene. The coated TiO_2_ films were immersed in 40 mM TiCl_4_ aqueous solution at 70 °C for 60 min in a preheated drying chamber. The FTOs were then washed vigorously with DI water and ethanol, and blow dried with an air stream. Finally, postheat conditions were set at 500 °C for 1 hr. In the Mxene-modified TiCl_4_ treatment, varying amounts of Mxenes (0.25 mg/mL and 0.37 mg/mL of Mxene) were added to the TiCl_4_ aqueous solution under the same processing conditions.

(d) Deposition of Mxene-modified mesoporous layer: In each of the aforementioned scaffolds, a mesoporous TiO_2_ layer was deposited through spin-coating by diluting the commercial paste (30-TS, G24 Power Ltd., Newport, UK, 30-TS/ethanol = 1/7, *w*/*w*) in ethanol at 6000 rpm for 25 s. Finally, the meso layer was annealed under ambient conditions through the following ramping heat treatment: 325 °C for 15 min, 375 °C for 10 min, 450 °C for 10 min, and 500 °C for 5 min. However, a conductive, mesoporous titania scaffold was achieved by adding a fixed amount of Mxene to the ethanol solution which was used to dilute the mesotitania paste. The varying compositions of all the prepared photoelectrodes with their labeling are presented in Table 1.

### 2.3. Materials Characterization

Crystallographic study of the synthesized samples was undertaken through the X-Ray diffraction (XRD) technique using a Bruker D8 ADVANCE system with a Cu tube source and a linear detector (LYNXEYE XE) (kalsruhe, Germany). Raman spectra were recorded on a Raman spectrometer (Renishaw InVia, Gloucestershire, UK), using 514.5 nm laser excitation. A TESCAN environmental scanning electron microscope (VEGA3 XMU, Brno, Czech Republic) with a LaB_6_ source and Oxford Aztec X-Max 50 EDS detector (High Wycombe, UK), were used for imaging and elemental mapping of the materials. The optical features of all prepared samples were studied using a UV-Vis spectrophotometer UV-2600i (Shimadzu corp., Kyoto, Japan). Current-voltage measurements were performed using an electrochemical workstation (Bio-Logic SAS SP-300, Seyssinet-Pariset, France) under solar illumination (100 mW cm^−2^), calibrated by a standard silicon solar cell (Model 15159, Abet Technologies, Inc. Milford, CT, USA). The applied electrochemical workstation was composed of an electrochemical cell with a varying applied voltage between the working electrode (synthesized films) and a reference electrode (Hg/HgO) in an aqueous 1.0M NaOH with a Pt wire as counterelectrode. 

## 3. Results and Discussion

### 3.1. (A) Mxene Powder Characterization

#### 3.1.1. Structural Analysis

**Figure 1:**
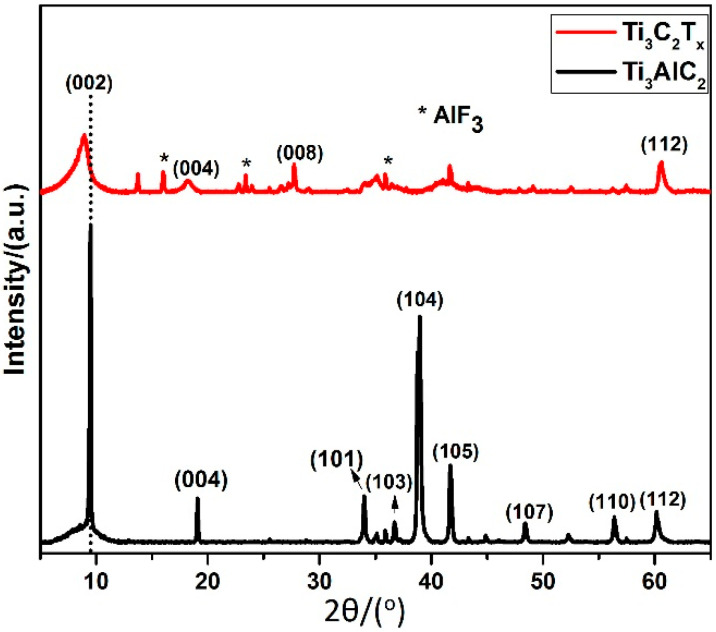
XRD patterns of Ti_3_AlC_2_ MAX precursor and as-synthesized Ti_3_C_2_T_x_ Mxene. Peaks marked with * are assigned AlF_3_ phase.

Figure 1 presents the XRD patterns of the Ti_3_AlC_2_ MAX powder used as starting material, and the etched Ti_3_C_2_T_x_ Mxene in HF acidic solution. Strong diffraction peaks of the starting material (Ti_3_AlC_2_) were observed at 2θ = 9.5°, 19.0^o^, 34.0°, 36.7°, 38.9°, 41.7°, 48.3°, 56.4°, and 60.1° [41,42]. In a broader context, and due to selective etching of Al layers from the starting material in a HF-assisted exfoliation process, the peaks were considerably shifted to lower angles (8.8°, and 18.2°) in the XRD spectrum of the synthesized Ti_3_C_2_T_x_ Mxene. This shift suggests an increased d spacing in the synthesized Ti_3_C_2_T_x_ Mxene (as shown in the SEM images below) compared to the starting Ti_3_AlC_2_ MAX phase. The increase in d spacing is due to structural expansion from etching and considerable substitution of Al with −F and −OH/=O terminating groups [43,44,45]. Moreover, the broadness and suppressed intensity of the peaks in the XRD pattern of Ti_3_C_2_T_x_ Mxene indicated less crystallinity and perturbed structural order in the sheets, which serves as evidence of successful treatment in HF acidic solution [46]. Along with these observations, the most intense peak at 2θ = 38.9° nearly disappeared in Ti_3_C_2_T_x_ Mxene, confirming the substantial removal of Al layers from Ti_3_AlC_2_ [47]. Furthermore, a new peak at 27.7° (008) was observed; this was due to the formation of Ti_3_C_2_(OH)_2_, in agreement with results reported by Li et al. [48]. Additionally, the appearance of other, less intense peaks (marked with *) was attributed to the presence of residual Al in the Ti_3_C_2_T_x_; this is difficult to remove completely with a 24 h soaking time and leads the formation of AlF_3_ [49,50,51]. These results suggest that the Ti_3_C_2_T_x_ surfaces were decorated with functional entities of −O and −F after HF treatment, as supported by the EDS spectra [52,53]. 

**Figure 2:**
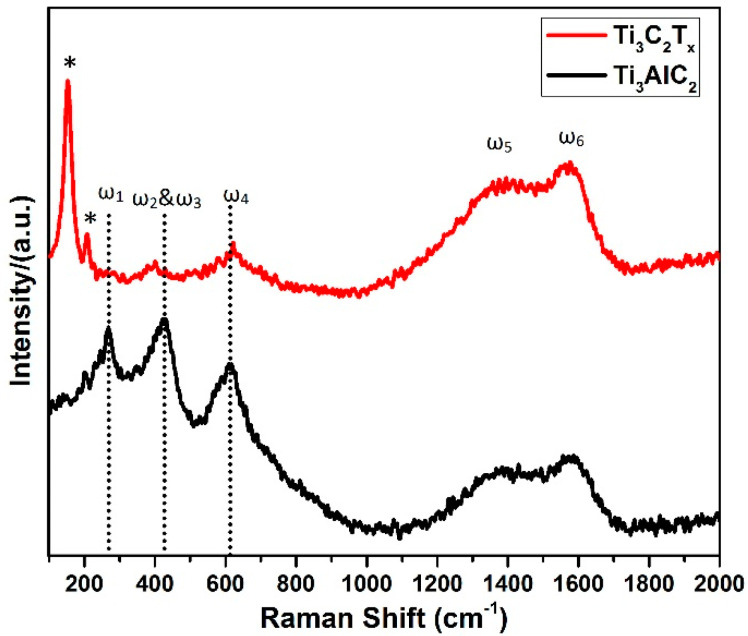
Raman Spectra of Ti_3_AlC_2_ MAX powder and Ti_3_C_2_T_x_ Mxene. Peaks marked with * are assigned to the out of plane vibrations of Ti and C atoms.

Figure 2 shows the Raman spectra of the Ti_3_AlC_2_ starting material in the MAX phase and the etched Ti_3_C_2_T_x_ Mxene. The peaks labelled as ω_1_, ω_2_ & ω_3_, ω_4_ in spectrum were located around 269, 423, and 613 cm^−1^ respectively. These peaks are key features of Ti_3_AlC_2_, and matched well with those reported in the literature, attributed to shear and longitudinal oscillations of Ti and the Al atoms [54,55,56,57]. Specifically, ω_1_ is associated with vibrations of Al; its disappearance in the of Mxene spectrum correlates with the substantial etching of Al atoms, resulting in the creation of a Mxene structure [58]. The broadening of the peaks in the Mxene spectrum, corresponds with a decrease in order, as expected by the exfoliation process; this was also observed in the XRD spectrum described above [59]. Additionally, another two broad peaks were observed in the range of 1000–1800 cm^−1^. These broad peaks were assigned to the D and G peaks of graphitic carbon, indicating the presence of carbon and disorder in the samples [60]. The increased intensity of these peaks showed that the surfaces had been exposed to more carbon. Graphitic carbon can greatly enhance the charge transfer characteristic of the carbide layer, which is highly advantageous for improved photoconversion efficiency of energy devices [61]. Both the G and D peaks were attributed to sp^2^ sites. However, the occurrence of a G-band is linked to the stretched C–C bond in carbonaceous substances, and is commonly associated with sp^2^ rings and chain architectures [62]. In contrast, the D peak occurs only in perturbed sp^2^ rings [63]. It is worth noting that the peak at 208 (marked with *) was attributed to the out-of plane vibrations of Ti and C atoms [50,64,65,66,67]. More prominently, there was an intense peak at around 151 cm^−1^. The high intensity of this peak could be due to the increased laser power, leading to the formation of oxidized Ti_3_C_2_ [53,68]. These results are supported by various reports in the literature, and can be taken to signify the successful synthesis of Ti_3_C_2_T_x_.

#### 3.1.2. Optical Features

**Figure 3:**
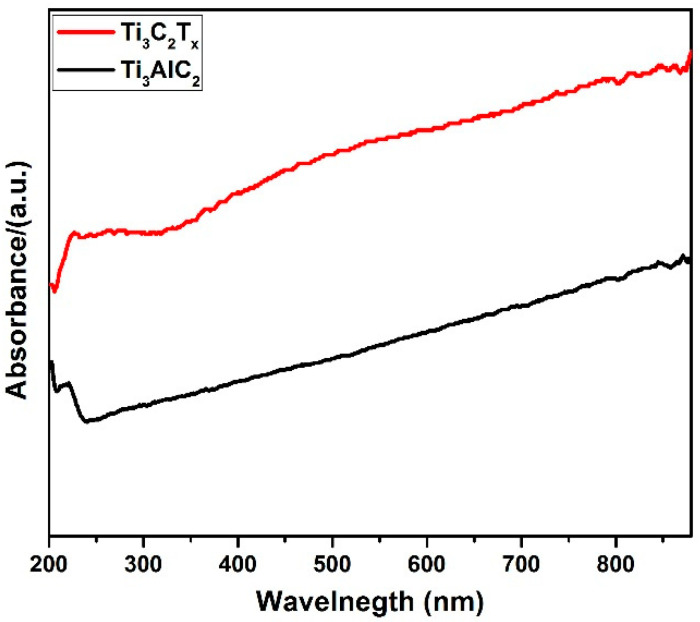
UV-Vis spectra of Ti_3_AlC_2_ MAX precursor and Ti_3_C_2_T_x_ Mxene.

The optical-response of the Ti_3_AlC_2_ MAX precursor and as-synthesized Mxene powder was investigated using the ultraviolet-visible (UV-Vis) absorption spectra. The Ti_3_AlC_2_ curve in Figure 3 and pristine Ti_3_C_2_T_x_ curve in Figure 3 indicate good optical response for an incident light range of 200–800 nm due to the black color of Ti_3_C_2_ with no clear absorption edge, indicating the metallic nature of Ti_3_C_2_ [69,70,71,72]. The broadness of these UV-Vis absorption bands are well-correlated with the previously reported optical features of Mxenes; this characteristic may be attributed to the localized surface plasmon resonance (LSPR) effect [73,74,75,76]. Surface plasmonic resonance effects (SP) are surface electronic oscillations which occur at the interlinked boundary between a metal and a dielectric material [77,78]. The surface effects become more pronounced with increased interlayer distance between the underlying Mxene sheets. Therefore, the absorption intensity of Mxene in curve b increased significantly in comparison with that of the Ti_3_AlC_2_ precursor phase [79,80,81]. 

#### 3.1.3. Morphological and Compositional Analysis

**Figure 4:**
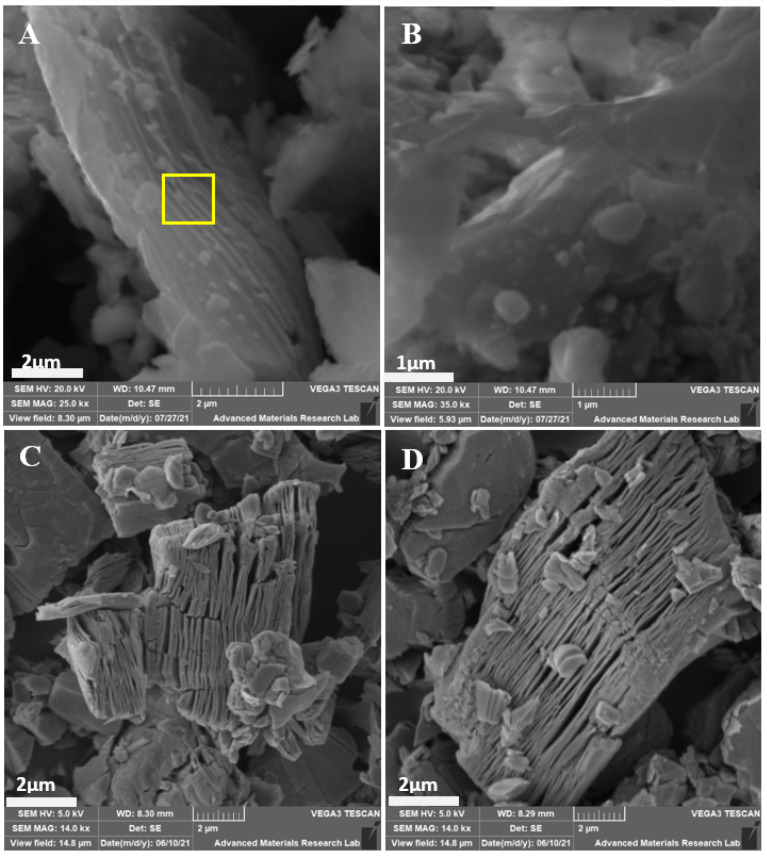
(**A**) SEM side view image of the Ti_3_AlC_2_ sheets; (**B**) SEM top view image of Ti_3_AlC_2_ sheets; (**C**,**D**) SEM side view image of Ti_3_C_2_T_x_.

SEM images make it possible to visually interpret the surface morphological features of synthesized materials. Figure 4A shows a side view of the Ti_3_AlC_2_ sheets, indicating a close-packed layer structure, as marked with a square box. Likewise, Figure 4B exhibits a typical top view of the Ti_3_AlC_2_ precursor, with lateral dimensions of around 4 µm. The EDS spectra and colorful elemental maps (shown in Figure S3 in supplementary material) of the Ti_3_AlC_2_ precursor exhibit the presence of Ti, C and Al, indicating the good purity level of the utilized Ti_3_AlC_2_ MAX phase precursor. Figure 4C shows the well-spaced and parallel layers in the Mxene sheets which resulted from HF acidic treatment. The resulting Ti_3_C_2_ had an interspacing of 0.38 µm, which is more than that found in bulk Ti_3_AlC_2_ (~0.16 µm). Such morphological transformations are similar to those observed in the literature [82], indicating the effective removal of Al layers from the precursor matrix. Figure 4D shows a few small spherical particulates with diameters of about ~0.53 µm, and tiny sheet-like fragments of lateral dimensions of 1.49 µm attached to the layered edges of the Ti_3_C_2_. These spherical species are AlF_3_ byproducts formed during the etching of Ti_3_AlC_2_ with HF [83], as evidenced by the XRD spectra and supported by the presence of fluorine and trace amounts of Al in the EDS spectra of the synthesized Ti_3_C_2_T_x_ Mxene; see Figure S4 in supplementary material. Due to etching of Ti_3_AlC_2_ in HF, the newly produced layered scaffold could be highly unstable due to its increased surface energy. Minimizing the surface energy the resulting layered structure increased its stability by anchoring the small fragments of Ti_3_C_2_ [84]. The HF etching of Ti_3_AlC_2_ in a controlled environment led to the successful production of exfoliated and lamellar Ti_3_C_2_ with Ti, C, O and F as the main components, as indicated by the EDS spectra shown in Figure S4. The amount of Al was considerably decreased compared to that in the Ti_3_AlC_2_ MAX phase precursor.

### 3.2. (B) Film Characterization 

#### 3.2.1. UV-Vis Absorbance Spectroscopy

**Figure 5:**
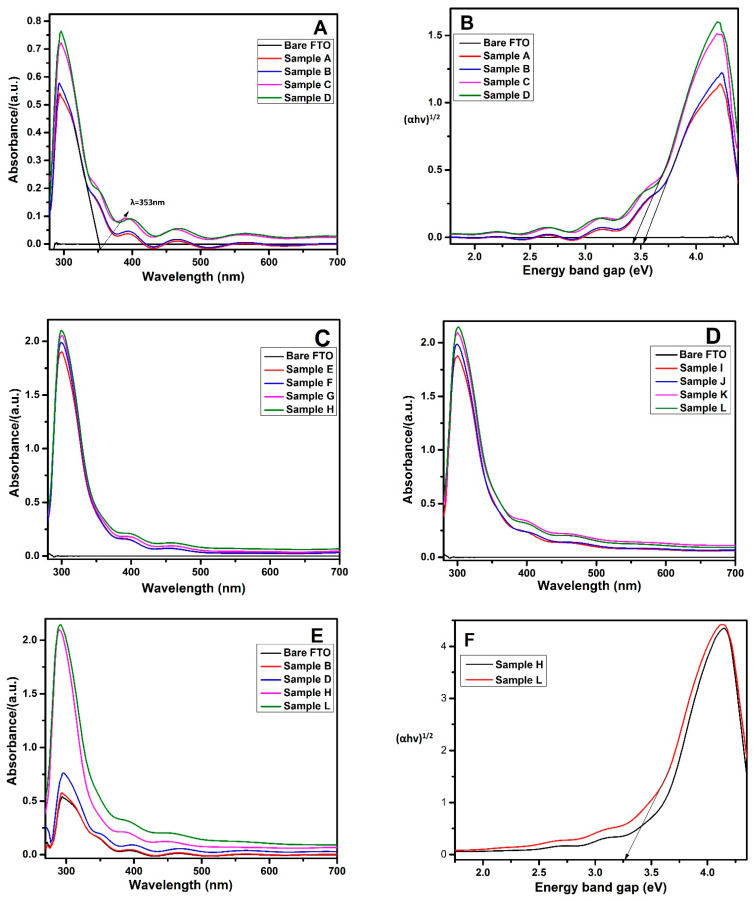
UV-Vis absorption Spectra (**A**,**C**–**E**) and tauc plot for corresponding energy band gap values (**B**,**F**).

The optical behavior of all the coated films on the FTO substrates was characterized using UV-Vis spectroscopy, as displayed in Figure 5. All the samples displayed good light-absorbing ability in the range of 350–700 nm, which leads to an enhancement of the opto-electronic device performance [85,86]. The main TiO_2_ absorption peak at a wavelength below 350 nm was clearly observed in all of the prepared film spectra, consistent with the published data [87,88,89]. Figure 5A shows a slight increase in the absorption intensity following TiCl_4_ treatment. On the other hand, a prominent increase in absorption intensity was observed due to the addition of Mxene in conventional TiCl_4_ treatment. This was attributed to the unique optical features of the Ti_3_C_2_ nanosheets presented in Figure 3. UV-Vis absorption became even more pronounced upon increasing the Mxene content. The improved optical response is also related to good film uniformity and dense coverage on the FTO substrates, as evidenced by XRD analysis [90]. Similarly, with mesoporous titania, a tremendous increase in the absorption peak intensity was observed for all samples, as shown in Figure 5C. Moreover, a significant increase in the absorption behavior within the visible region was observed (Figure 5D) in the case of Mxene functionalized mesoporous titania. A clear difference in the photon absorption intensity due to mesoporous layer deposition is prominently visible in Figure 5E. The figure also shows a pronounced increase in light absorption efficiency within the visible region, as shown in the green curve, due to the addition of Mxene in the final deposited mesoporous layer. 

Band alignment and band gap are other important characteristics that describe the optical features of a photocathode. The energy band gap values of all of the prepared films were determined through Tauc’s method, given in Equation (4) [91].

(4) $${\left(\alpha \mathrm{hv}\right)}^{n}=A\;\left(\mathrm{hv}-E_{g}\right)$$

where A is a constant, Eg is the band gap energy and n is a number specifying the transition process. The value n = 2 is assigned for a direct transition and n = ½ for an indirect transition [92]. The most widely used methodology to determine Eg involves plotting (αhv)^n^ against photon energy hv. The plot of (αhv)^1/2^ versus photon energy (hv) is shown in Figure 5B,F. The calculated energy band gap value for TiO_2_:Mxene film was 3.51 eV (see Figure 5B), which was then downshifted to 3.44 eV in Mxene-modified TiCl_4_ treated TiO_2_:Mxene. A further reduction in energy band gap, i.e., down to 3.26 eV, was observed in the Mxene functionalized mesoporous titania layer (Figure 5F) due to a larger Mxene content. The improvements in the optical and electronic properties of Mxene functionalized mesoporous titania are in agreement with the obtained IV characteristics of films, as shown below.

#### 3.2.2. Structural Analysis of the Mxene Functionalized Mesoporous Titania Layer

**Figure 6:**
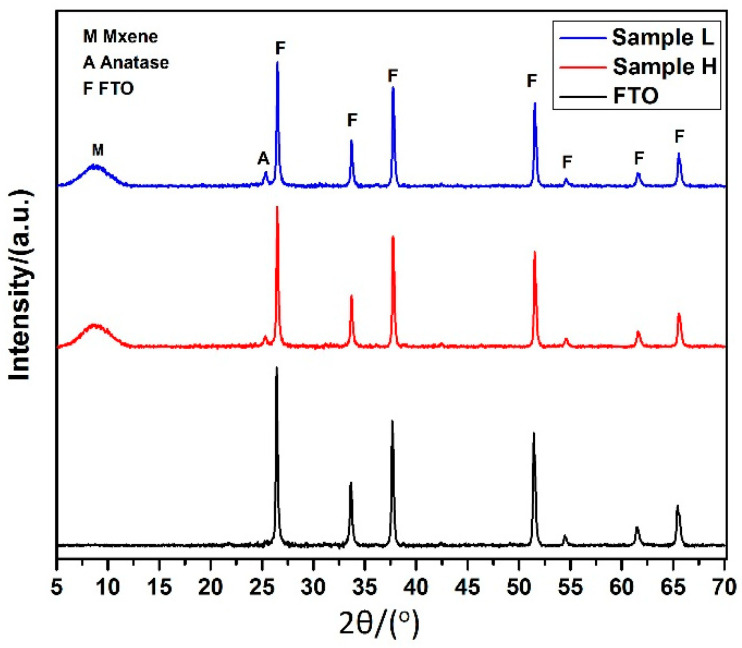
XRD spectra of bare FTO, Sample H, and Sample L.

The structural, phase identification and crystallinity of the deposited films were investigated by XRD. Figure 6 shows the XRD patterns of FTO substrates: (a) Sample H, i.e., Glass/FTO/cTiO_2_ + Mx/TiCl_4_ + 3%Mx/mTiO_2_; and (b) Sample L, i.e., Glass/FTO/cTiO_2_ + Mx/TiCl_4_ + 3%Mx/mTiO_2_ + Mx. The figure demonstrates that the fabricated films exhibited a tiny single anatase peak of TiO_2_ (101) phase at 2θ = 25.2° and a broad peak at 2θ = 8.6° (002) for Mxene. The peak positions for both the Mxene and Anatase phases were in agreement with those reported in the literature [93,94,95,96]. For the sake of comparison, the XRD pattern of the bare FTO is shown in Figure 6; it reveals several peaks, indexed at 2θ = 26.49°, 33.6°, 37.69°, 51.48°, 61.44°, and 65.48°, with identified planes (110), (101), (200), (220), (221), and (301) respectively [97]. Interestingly, due to the Mxene content following TiCl_4_ treatment and the mesoporous layer, the peak of TiO_2_ became more prominent, supporting the increased crystallinity and well-grown anatase phase of TiO_2_. Heat treatment of Mxene in open air at high temperature reinforced the formation of the anatase phase [98]. Additionally, the characteristic peaks of the FTO substrate were significantly suppressed in the as-prepared films. The anatase phase of TiO_2_ has been reported to provide better electron conduction with a greater absorption coefficient [99]. The improved crystallinity in the films is advantageous for photoelectric conversion activity, as well developed crystalline attributes ensure enhanced photo stability for efficient conduction of phototriggered charge carriers [100]. These factors contributed to enhance the photo assisted electrical conductance of the fabricated films. 

**Figure 7:**
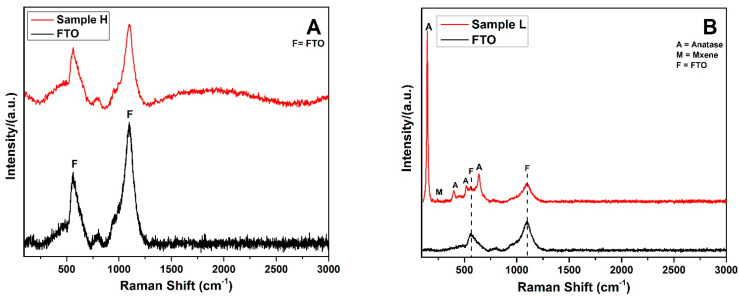
(**A**) Raman Spectra of FTO, Sample H; and (**B**) Raman Spectra of Sample L.

The obtained structural features were further investigated by Raman spectroscopy. Figure 7A presents the Raman spectra of bare FTO (a) and Sample H: Glass/FTO/cTiO_2_ + Mx/TiCl_4_ + 3%Mx/mTiO_2_ (b), respectively. The characteristic FTO peaks at 562 and 1094 cm^−1^ [101] were greatly reduced in the deposited film. The slightly increased intensity in the range of 1500 cm^−1^–2500 cm^−1^ in the red curve in Figure 7A was attributed to disordered graphitic carbon due to the Mxene content [102]. Similar Raman results are shown in Figure 7B for the synthesized film Sample L Glass/FTO/cTiO_2_ + Mx/TiCl_4_ + 3%Mx/mTiO_2_ + Mx (b) and for bare FTO (a). Due to the addition of Mxene in the TiCl_4_ treatment and in the mesoporous layer, the Raman peaks of the anatase phases at 401, 522, and 643 cm^−1^ (B1g), (A1g), and (Eg) vibrations respectively were clearly visible [103]. These results are in agreement with the XRD results reported above. A small narrow peak at 242 cm^−1^ was attributed to the in-plane (shear) modes of the Ti which is present in Mxene [104]. Along with that, the characteristic FTO peaks, marked F in Figure 7B, decreased significantly in the final synthesized film.

#### 3.2.3. Morphological Analysis

**Figure 8:**
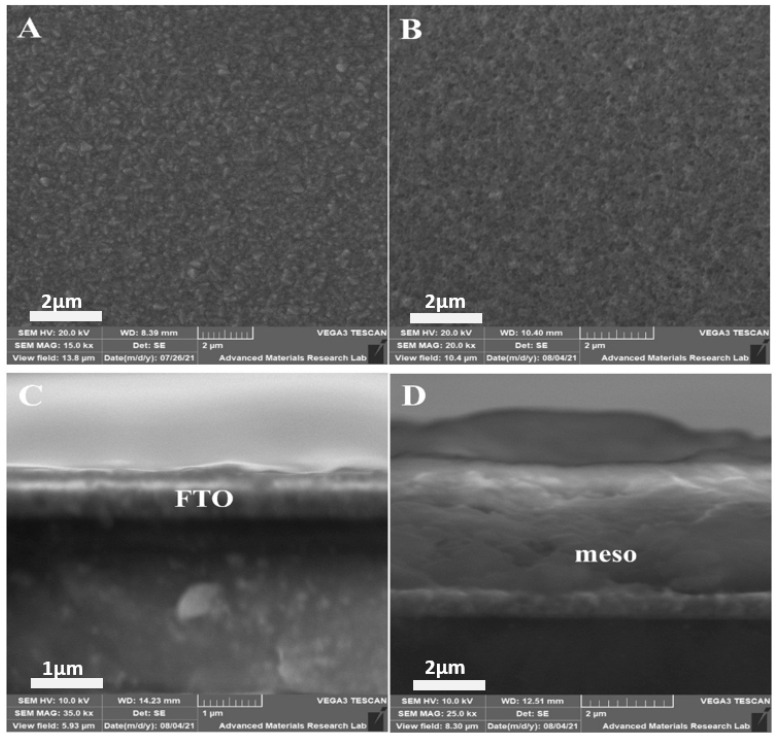
Front view SEM micrographs of Sample D (**A**); Front view SEM micrographs of Sample L (**B**); Cross-sectional side view SEM micrographs of Glass/FTO (**C**); Cross sectional top view SEM micrographs of Sample L (**D**).

The morphological features of the synthesized films were analyzed through SEM micrographs, as shown in Figure 8A–D. The compact morphology of TiO_2_:Mx without any obvious pinholes is clearly noticeable in Figure 8A. The well-grown crystallites of 0.5 µm grain size provided better surface coverage. The flake-like structures of Mxene sheets were prominent, along with spherical particles of TiO_2_. Such significant interactions between Mxene flakes and TiO_2_ spheres in crack-free films due to subsequent Mxene-modified TiCl_4_ treatment and postdeposition annealing are favorable for quick charge segregation [105]. The mesoporous TiO_2_ layer shown in Figure 8B was composed of an ordered mesoporous structure consisting of various spherical nanograins with good porous features. Additionally, eximious interconnectivity was present throughout the structure, yielding better light absorption efficiency (as evidenced by UV-Vis spectroscopy) due to the high surface area and small particle size of the spherical nanograins [106]. The excellent interparticle connectivity improved the conductance of the photogenerated electrons to the FTO contact surface [99]. A cross sectional side view of bare FTO is shown in Figure 8C. Likewise, the cross sectional top view of the stacked architecture, composed of glass/FTO/compact TiO_2_:Mx layer/mesoporous TiO_2_:Mx, is presented in Figure 8D. The top view exhibits an optimally thick, mesoporous layer with slightly rough surface features. The dense morphology of the underlying compact layer and high porosity with excellent interconnectivity of the m-TiO_2_ conductive scaffold contributed significantly to the enhanced photovoltaic properties of the photoelectrode in terms of increased charge carrier generation with a suppressed recombination rate [107].

#### 3.2.4. Photo-Current Measurements

**Figure 9:**
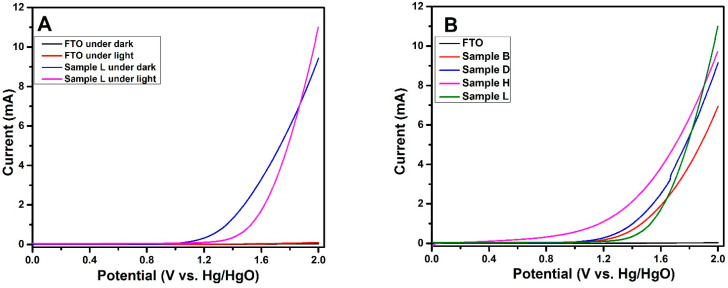
(**A**) IV curves of FTO and Sample L under darkness and illumination; (**B**) Phototriggered IV curves for FTO, Sample B, Sample D, Sample H, and Sample L.

The photocurrent features of the fabricated electrodes were evaluated by linear sweep voltammetry. The scanning rate for the linear sweep voltammetry measurements were set at 20 mV s^−1^. These photocurrent attributes were measured under 1 sun (100 mWcm^−2^ AM 1.5) and under dark conditions, as displayed in Figure 9A. All the synthesized thin films were found to be photoactive (presented in Figure 9B) due to the good crystallinity of their anatase phase. However, each film showed different magnitudes of photocurrent, depending on the material characteristics due to different Mxene contents. Figure S5 shows a schematic representation of the electrochemical setup used.

Upon illuminating, the photoelectrode with the highest Mxene content in the TiCl_4_ solution, as well as in mesoporous titania (sample L), exhibited the highest photocurrent, as shown in Figure 9B. Two possible factors were responsible for the enhanced photocurrent: (a) better electron–hole separation due to significant interaction between Mxene and TiO_2_ particles; and (b) the surface plasmon resonance effect of Mxene (as evident in Figure 3) causing increased photoabsorption. Moreover, the good crystallinity in the mesoporous titania scaffold minimized the charge annihilation process. Mxene acts as a conducting medium for increased electrical conductance within the mesoporous titania network. In this study, excellent photocurrents were recorded with Mxene-modified, mesoporous treated samples (Sample L, Figure 9B), in comparison with the films without mesoporous layer deposition (Sample B of Figure 9B). These electrical properties are supported by the optical behavior of the films, as shown in Figure 5, with comparably high absorption intensity with a mesoporous titania layer. Consequently, a larger number of absorbed photons led to increased generation of photoexcited charge carriers, resulting in more photocurrent. However, the newly developed photoelectrodes could be further optimized by varying the amount of Mxene added to the TiO_2_ scaffold, by modulating the morphology of the Mxene sheets for enhanced opto-electric features, and by adopting alternate coating procedures, such as spray pyrolysis, for large-scale production.

## 4. Conclusions

In summary, a novel combination of Mxene functionalized mesoporous TiO_2_ photoelectrodes is reported. The inherent features of mesoporous TiO_2_ scaffolds were improved by inducing conductive species in order to reduce the presence of electronic trap sites for enhanced electron conductivity. Therefore, the Mxene contents were successfully added to pristine TiO_2_ in a controlled fashion through multistep spin coating process. Structural investigations revealed an increased crystallinity of the anatase phase with increased Mxene content in the fabricated films. Moreover, Mxene-modified TiCl_4_ treatment and postdeposition annealing conditions were found to favor the growth of crack-free films for quick charge segregation. We also reported enhanced photocurrents for photoelectrodes with higher Mxene contents due to significant improvements in absorption efficiency in the visible region, as verified by UV–Vis absorption spectroscopy. Furthermore the minimized charge annihilation process supported better electron–hole separation because of the significant interaction which occurred between Mxene and TiO_2_ particles.

## Figures and Tables

**Table 1:** List of the compositions of all prepared photoelectrodes.

No of Samples	Photoelectrode Architecture	Photoelectrode Labelling
1	Glass/FTO/TiO_2_:Mx	Sample A
2	Glass/FTO/TiO_2_:Mx/TiCl_4_	Sample B
3	Glass/FTO/TiO_2_:Mx/TiCl_4_:0.25 Mx	Sample C
4	Glass/FTO/TiO_2_:Mx/TiCl_4_:0.37 Mx	Sample D
5	Glass/FTO/TiO_2_:Mx/mTiO_2_	Sample E
6	Glass/FTO/TiO_2_:Mx/TiCl_4_:mTiO_2_	Sample F
7	Glass/FTO/TiO_2_:Mx/TiCl_4_:0.25 Mx/mTiO_2_	Sample G
8	Glass/FTO/TiO_2_:Mx/TiCl_4_:0.37 Mx/mTiO_2_	Sample H
9	Glass/FTO/TiO_2_:Mx/mTiO_2_+Mx	Sample I
10	Glass/FTO/TiO_2_:Mx/TiCl_4_/mTiO_2_+Mx	Sample J
11	Glass/FTO/TiO_2_:Mx/TiCl_4_:0.25Mx/mTiO_2_+Mx	Sample K
12	Glass/FTO/TiO_2_:Mx/TiCl_4_:0.37Mx/mTiO_2_+Mx	Sample L

## Data Availability

Not applicable.

## Supplementary Materials

The following are available online at https://www.mdpi.com/article/10.3390/ma14216292/s1, Figure S1: Schematic representation of stages involved in the synthesis of Mxene sheets; Figure S2: Stages involved in the fabrication of Mxene modified Mesoporous Titania Photoelectrodes; Figure S3: The EDS spectra and colorful elemental maps of Ti_3_AlC_2_ precursor exhibit the contents of Ti, C and Al elements indicating good purity level of the utilized Ti_3_AlC_2_ MAX phase as precursor; Figure S4: Successful production of the exfoliated and lamellar Ti_3_C_2_ due to HF etching of Ti_3_AlC_2_ in a controlled environment, with Ti, C, O and F as the main components indicated in EDS spectra; Figure S5: A pictorial view of Mxene functionalized mesoporous TiO_2_ layer working as photoelectrode under light illuminations.
