# Supplementary material for: Investigation and Optimization of Mxene Functionalized Mesoporous Titania Films as Efficient Photoelectrodes
Source: Materials (Basel). 2021 Oct 22;14(21):6292. doi: 10.3390/ma14216292 (PMC8585131; doi:10.3390/ma14216292)
Supplement: Supplementary file 1 [file materials-14-06292-s001.zip › materials-1414990-supplementary.pdf]

materials

Supporting Information

Investigation and Optimization of Mxene functionalized Mesoporous Titania Films as Efficient Photoelectrodes

Anum Iqbal[1] and Nasser M. Hamdan[2],*

[1]Material science and Engineering Program, The American University of Sharjah
[2]Physics Department,
The American University of Sharjah, Sharjah 26666, United Arab Emirates;
*Corresponding author Email: (nhamdan@aus.edu)

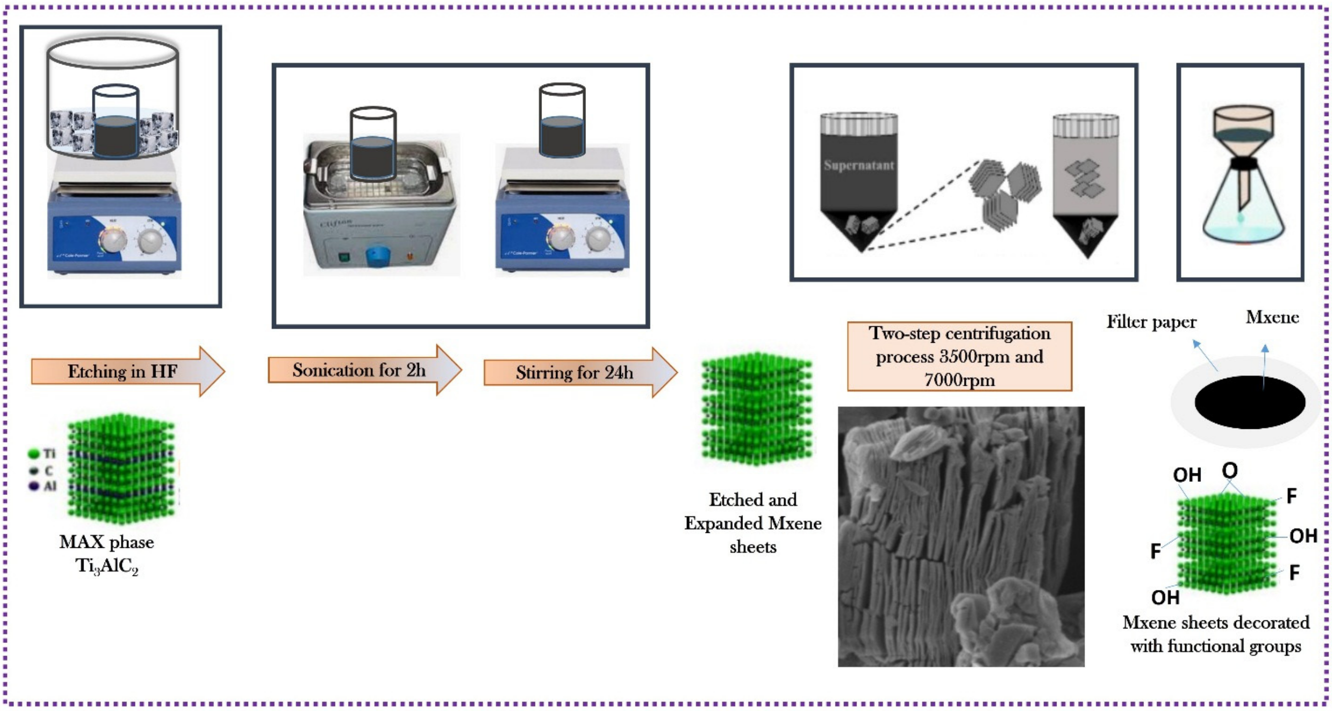

Figure S1. Schematic representation of the stages involved in the synthesis of Mxene sheets.

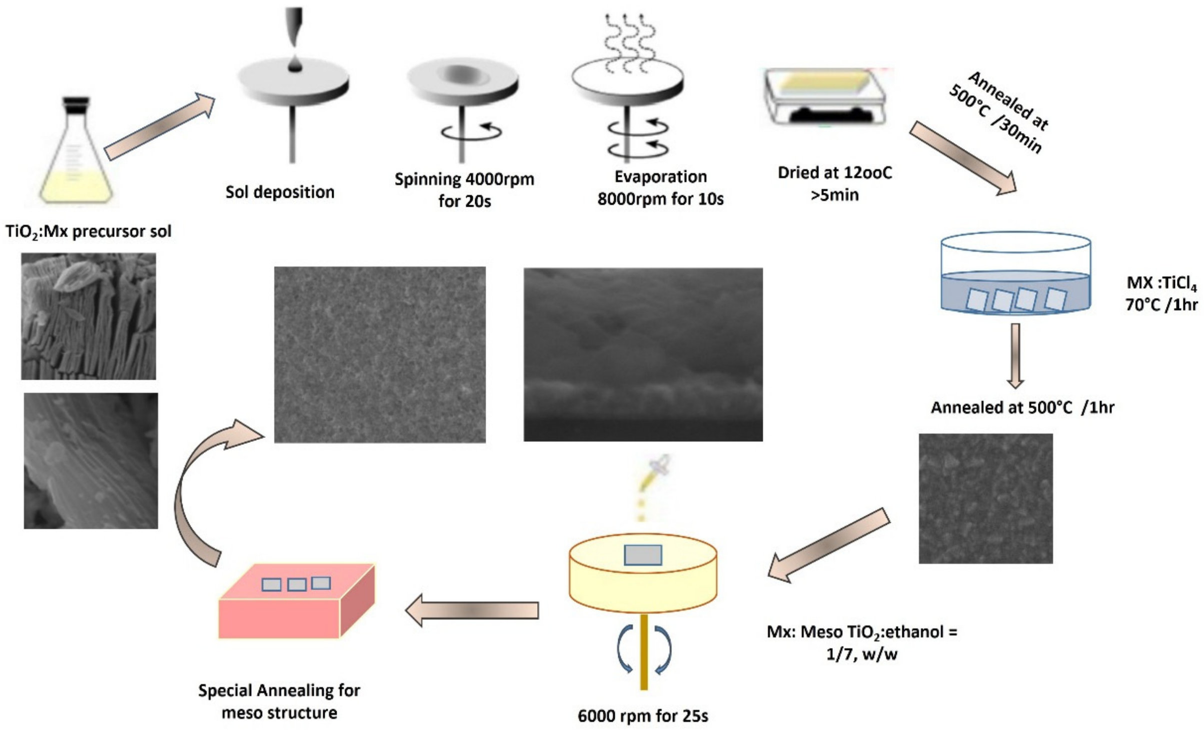

Figure S2. Stages involved in the fabrication of Mxene modified Mesoporous Titania Photoelectrodes.

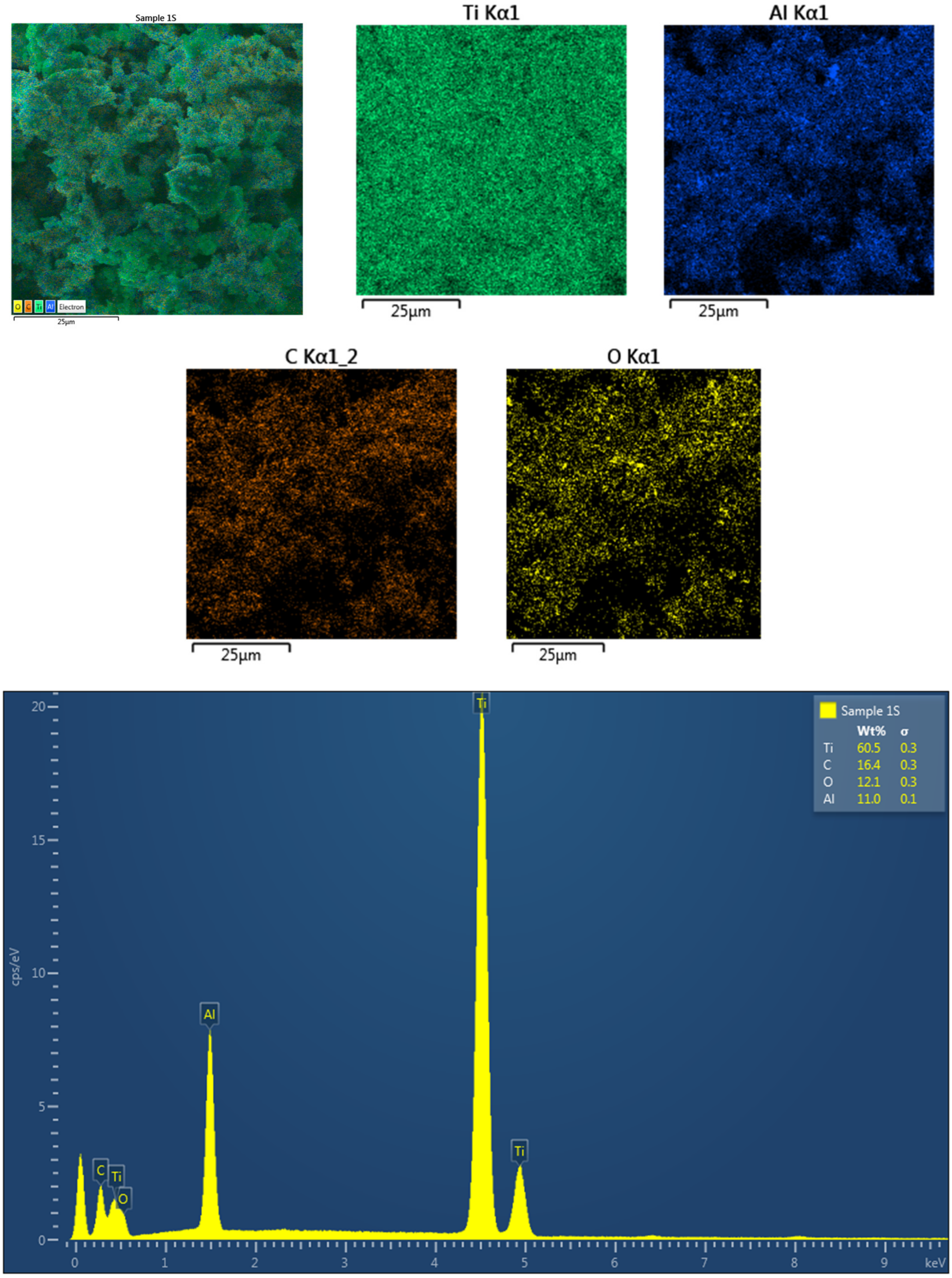

Figure S3. The EDS spectra and colorful elemental maps of Ti_3AlC_2 precursor exhibit the contents of Ti, C and Al elements indicating good purity level of the utilized Ti_3AlC_2 MAX phase as precursor.

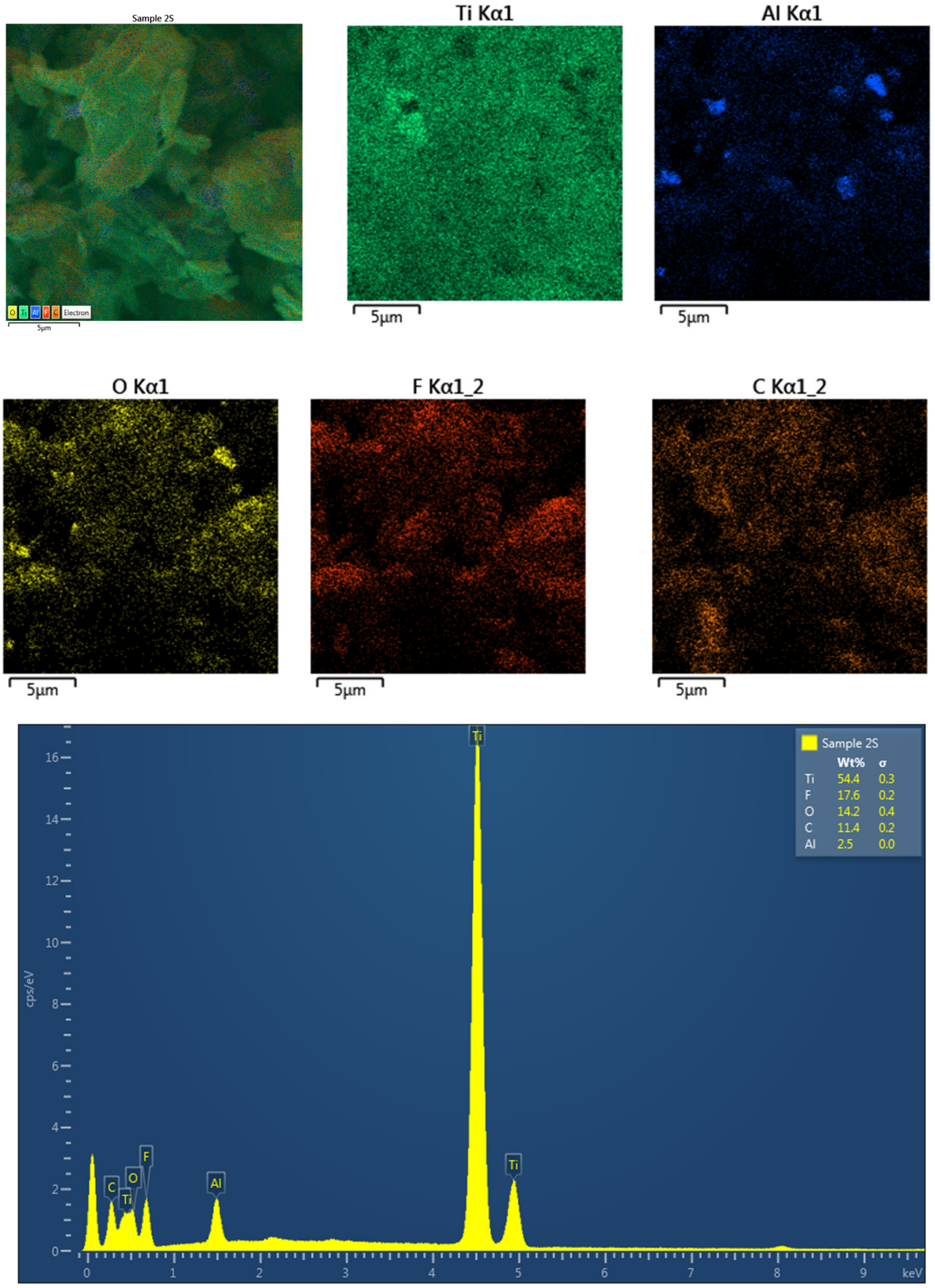

2

Figure S4. Exfoliated and lamellar Ti_3C_2 post HF etching of Ti_3AlC_2 in a controlled environment, with Ti, C, O and F as the main components indicated in the elemental maps and EDS spectra. The presence of few Al spots indicates the presence of unreacted AlF_3, which was also confirmed from XRD results shown in fig 1 of the manuscript.

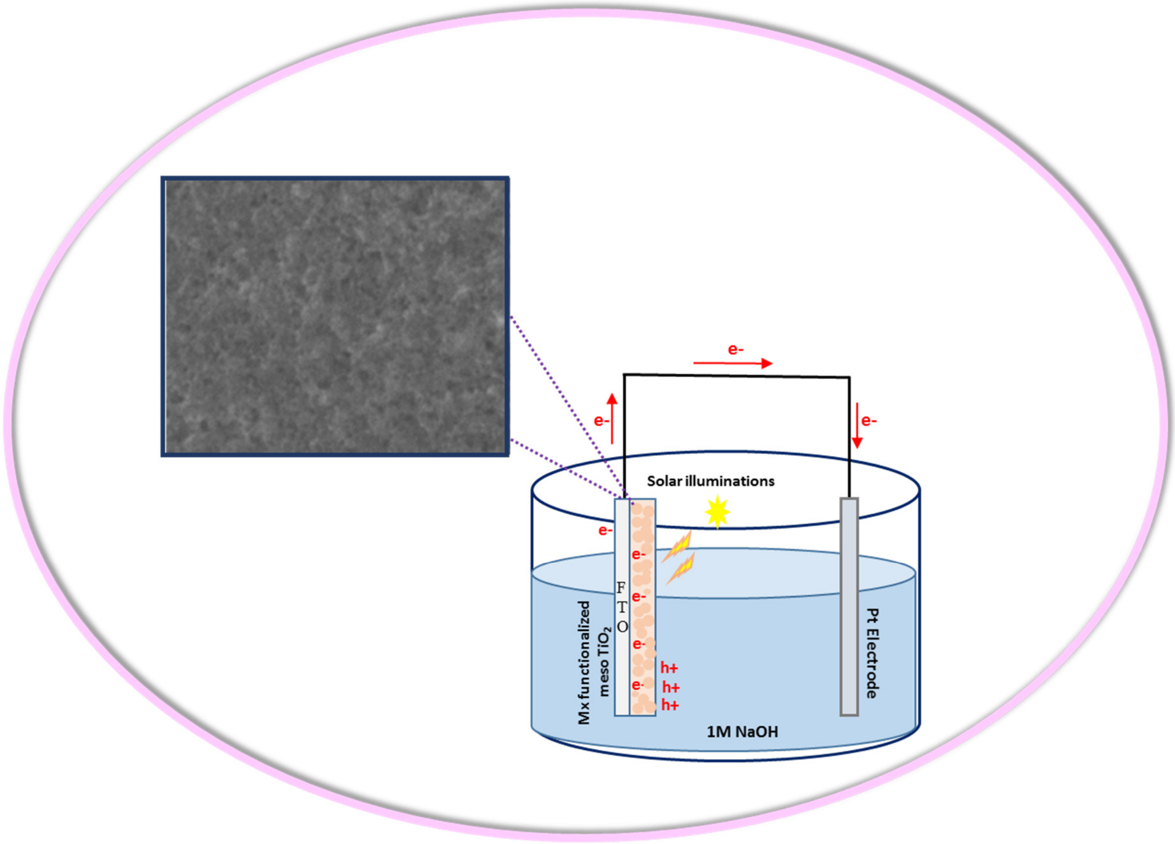

Figure S5. A pictorial view of Mxene functionalized mesoporous TiO_2 layer working as photoelectrode under light illuminations.
